# Minimal detectable change of gait and balance measures in older neurological patients: estimating the standard error of the measurement from before-after rehabilitation data thanks to the linear mixed-effects models

**DOI:** 10.1186/s12984-024-01339-4

**Published:** 2024-04-02

**Authors:** Antonio Caronni, Michela Picardi, Stefano Scarano, Viviana Rota, Giacomo Guidali, Nadia Bolognini, Massimo Corbo

**Affiliations:** 1 Department of Neurorehabilitation Sciences, Ospedale San Luca, IRCCS, Istituto Auxologico Italiano, Milan, 20149 Italy; 2 Department of Biomedical Sciences for Health, Università degli Studi di Milano, Milan, 20133 Italy; 3 Department of Neurorehabilitation Sciences, Casa di Cura Igea, Milano, 20144 Italy; 4 Department of Psychology and NeuroMI, University of Milano-Bicocca, Milano, 20126 Italy; 5 IRCCS Istituto Auxologico Italiano, Via Giuseppe Mercalli, 28, Milano, 20122 MI Italia

**Keywords:** Measurement error, Standard error of the measurement, Minimal detectable change, Gait assessment, Balance assessment, Falling risk, Linear mixed-effects models, Inertial measurement units

## Abstract

**Background:**

Tracking gait and balance impairment in time is paramount in the care of older neurological patients. The Minimal Detectable Change (MDC), built upon the Standard Error of the Measurement (SEM), is the smallest modification of a measure exceeding the measurement error. Here, a novel method based on linear mixed-effects models (LMMs) is applied to estimate the standard error of the measurement from data collected before and after rehabilitation and calculate the MDC of gait and balance measures.

**Methods:**

One hundred nine older adults with a gait impairment due to neurological disease (66 stroke patients) completed two assessment sessions before and after inpatient rehabilitation. In each session, two trials of the 10-meter walking test and the Timed Up and Go (TUG) test, instrumented with inertial sensors, have been collected. The 95% MDC was calculated for the gait speed, TUG test duration (TTD) and other measures from the TUG test, including the angular velocity peak (ω_peak_) in the TUG test’s turning phase. Random intercepts and slopes LMMs with sessions as fixed effects were used to estimate SEM. LMMs assumptions (residuals normality and homoscedasticity) were checked, and the predictor variable ln-transformed if needed.

**Results:**

The MDC of gait speed was 0.13 m/s. The TTD MDC, ln-transformed and then expressed as a percentage of the baseline value to meet LMMs’ assumptions, was 15%, i.e. TTD should be < 85% of the baseline value to conclude the patient’s improvement. ω_peak_ MDC, also ln-transformed and expressed as the baseline percentage change, was 25%.

**Conclusions:**

LMMs allowed calculating the MDC of gait and balance measures even if the test-retest steady-state assumption did not hold. The MDC of gait speed, TTD and ω_peak_ from the TUG test with an inertial sensor have been provided. These indices allow monitoring of the gait and balance impairment, which is central for patients with an increased falling risk, such as neurological old persons.

**Trial registration:**

NA.

**Supplementary Information:**

The online version contains supplementary material available at 10.1186/s12984-024-01339-4.

## Background

In medicine, it is extremely important to determine whether a patient has worsened significantly, for example because of disease progression, or improved significantly, such as after rehabilitation [1, 2].

The Minimal Detectable Change (MDC) represents the smallest change in a measured variable not attributable to measurement error [2, 3]. The MDC is a confidence interval (CI), most commonly a 95% CI, built around a null difference between two measures. In front of a difference between measures exceeding the 95% MDC, one can be 95% confident that this difference is not due to a mere measurement error.

Central to the MDC calculation is the estimation of the Standard Error of the Measurement (SEM). As the standard deviation of the measurement error [4], SEM quantifies the precision of a single measure [5]: the lower the SEM, the smallest the measurement error and the better (i.e., the more precise) the measure. In turn, the lower the SEM, the lower the MDC since the MDC is the SEM multiplied by a constant.

Various neurological diseases, such as stroke, peripheral neuropathy of the lower limbs and Parkinson’s disease, impair gait and balance in older adults [6]. This gait and balance impairment reduces the person’s independence [7] and increases the risk of falling [8, 9]. Therefore, it is critical to identify people with impaired mobility, estimate the amount of this impairment, administer treatments that may improve it effectively, and monitor the impairment over time.

In this context, it is not surprising that numerous gait and balance measures have been developed so far. Among the gait measures, it is worth mentioning the gait speed, which has been shown to predict several adverse events (including falls, hospitalisation and mortality) [10]. Among the balance measures, i.e. mobility measures reflecting the ability to not fall, are those from the Timed Up and Go (TUG) test [11, 12]. In this test, examinees are asked to stand up from a chair, walk straight for a few meters, turn, walk back to the chair and sit down. A longer TUG test duration is associated with an increased risk of falls in older adults [6, 13].

Given the importance of adequately monitoring gait and balance in time, the MDC has been calculated for gait and balance measures, and even systematic reviews are available on this topic. However, these same reviews point out that the MDC is the least frequently assessed psychometric property of mobility measures [14], encouraging further research on its adoption in different clinical conditions, ranging from knee osteoarthritis [15] to stroke [16].

Conventionally, dedicated test-retest experiments are run to estimate SEM. Individuals are measured twice (i.e., tested and retested), with an interval between assessment sessions short enough so that no modification of the patient’s status occurs (e.g. there is no disease progression) but sufficiently delayed so that to prevent test recall. Central to these experiments is the steady-state assumption, i.e., the measured variable’s quantity does not change between the two assessment times.

Three methods are commonly used for estimating SEM from test-retest, steady-state experimental designs [5, 17], namely. SEM can be:

1. Derived from a reliability index like the intraclass correlation coefficient (ICC);
2. Estimated by the limits of agreement of a Bland-Altman analysis;
3. Calculated as the square root of the mean square error term from an Analysis of Variance (ANOVA) model.

Each approach has pros and cons, but it is interesting to note that the first one, likely the most commonly used, has been harshly criticised to the point that some psychometricians discourage its application [17].

For example, since different reliability indices are available (e.g. a family of ICCs [18, 19]), the choice of the reliability index can affect the SEM size [5] and comparing the SEM of different studies can be challenging. On the contrary, this limitation does not apply to the estimation of SEM from ANOVA residuals, making this method recommendable [5].

Recently a novel method for estimating the SEM, which applies the linear mixed-effects models (LMMs) to data collected before and after treatments, has been proposed [20–24]. Compliance with the steady-state assumption is hard to defend when patients receive treatment between the two assessment sessions. In this scenario, LMMs come to the rescue. With this approach, time and treatment effects are incorporated into the model and accounted for: statistical modelling creates a steady state in the analysis phase, rather than in the experimental phase, so that repeated measures with a different mean structure can be used to quantify the measurement error [20]. Similarly to the above-mentioned ANOVA analysis, the SEM is eventually estimated through variance decomposition and from the residual variance in the first place.

On these bases, the present study aims to calculate the SEM and the 95% MDC of common measures from the walking and TUG tests administered to older neurological patients. To this aim, SEM is estimated from LMMs run on data from older neurological patients collected before and after rehabilitation, which included balance training. Emphasis is placed on simple assessment procedures and low-tech measures that can be easily implemented in the normal clinic and research setting.

## Methods

This observational, longitudinal study analyses data from a more extensive project, approved by the local ethics committee (Comitato Etico Milano Area 2; 568_2018bis) on fall risk assessment in neurological patients at discharge from rehabilitation. The primary study’s results on fall risk have been recently published [25].

Inclusion criteria were:

- Age over 65 years;
- Affected by one of the following neurological syndromes: hemiparetic gait secondary to a stroke, peripheral neuropathy of the lower limbs or parkinsonism secondary to a vascular encephalopathy or Parkinson’s disease.
- Informed consent to participate in the study.

With respect to the exclusion criteria:

- Presence of two neurological diagnoses (e.g. hemiparesis and Parkinson’s disease);
- Inability to complete the TUG test and the 10 m walking test without touching assistance on admission or discharge;
- Completing any of the mobility tests using an assistive device (e.g. a cane, a walking frame) on admission or discharge;
- Completing the TUG test in more than 30 s on admission or discharge;
- Severe visual impairment or hearing loss.

### Assessment

**Fig. 1:**
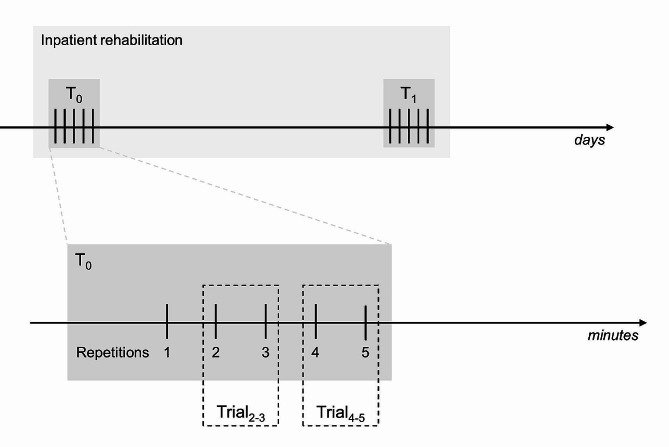
Time course of the mobility assessment. Gait and balance have been assessed in two assessment sessions, T_0_ and T_1_ (dark grey boxes), the first at the beginning and the second at the end of the participants’ inpatient rehabilitation stay (light grey box). Five repetitions of the 10 m walking and TUG tests have been collected in each session. Trial_2 − 3_ is constituted of repetitions 2 and 3 while Trial_4 − 5_ by repetitions 4 and 5. Measures of the variable of interest from a single test repetition are referred to here as “metric” (e.g. the five repetitions of, say, the 10 m walking test return five metrics of gait speed). Participants’ measures are given by the mean of two metrics (e.g. participants’ gait speed measures from Trial_2 − 3_ are the mean of the gait speed metrics from repetitions 2 and 3) or by the median of all five repetitions. Horizontal arrows: time, expressed in days and minutes for the upper and lower drawings, respectively

Participants completed two assessment sessions, the first at the beginning (T_0_) and the second at the end (T_1_) of the rehabilitation program (Fig. 1). In each session, a detailed balance and gait assessment was administered. In the current study, only data from the 10 m walking test and the TUG test are reported.

A physiotherapist or an occupational therapist administered both tests, and both the 10 m walking test and the TUG test were repeated five times in each session. Participants completed the TUG test with an inertial measurement unit (mHT-mHealth Technologies, Bologna, Italy) attached to their lower trunk.

In the 10 m walking test [26], participants were asked to walk straight while a clinician measured with a stopwatch the time taken to walk the central six meters of a 10 m long trajectory.

The conventional three m TUG test [11] was administered as follows. Participants were asked to wait for a go signal from the experimenter and then stand up from a chair, walk in a straight line, turn around a traffic cone, return to the chair and sit down. Trunk acceleration and angular velocity along the three axes were recorded by the inertial sensors. In addition, the experimenter measured the TUG test duration (TTD) with a stopwatch.

The 10 m walking and TUG tests were completed at the patient’s comfortable speed.

In our previous works, the five test repetitions were analysed individually, mobility metrics^1^ were collected from each, and their median value was used as the patient’s mobility measure (e.g. [12, 27–29]). Here, instead, persons’ measures were obtained from two repetitions, a full-fledged shortened version of the five repeats mobility tests. This choice was taken to calculate more accurate LMMs for SEM estimation (i.e. maximal models [30] with random intercepts and slopes, see below). As a preliminary step, the measurement accuracy of the reduced tests was compared to our previous measurement reference (i.e., the five repeats tests).

The gait speed (m/s) was calculated for each 10 m walking test repetition, and from each TUG test repetition the following were collected:

1. TUG test duration (TTD, s),
2. Sit-to-walk (STW) duration (s),
3. Turning duration (i.e. the duration of the first turning phase; s),
4. Peak angular velocity along the vertical axis during turning (ω, °/s).

For the reduced versions of the walking and TUG tests, the first of the five repetitions was considered a “test ride” (e.g. the patient gets confidence with the test set), thus being discarded.

Metrics from repetitions 2 and 3 (which together form Trial_2 − 3_) are averaged, and these average values are called Trial_2 − 3_ measures. Similarly, metrics from repetitions 4 and 5 are averaged and referred to as Trial_4 − 5_ measures.

It is worth stressing that in the reduced versions of the walking and TUG test set up here, a *single* participant’s measure (e.g., the participant’s gait speed and TTD) comes from *two* test repetitions. Hence, in each session, two participants’ measures were collected. As explained below, Trial_4 − 5_ is used to assess the stability of the Trial_2 − 3_ measures.

The inertial measurement unit software automatically returned the STW, turning duration, and ω metrics for each test repetition [31].

### Statistics

#### Assessing the agreement between the reduced and the five-repeat mobility tests

The agreement between the measures from the reduced versions of the walking and TUG tests and those from the complete tests (i.e. the five-repeat tests) was assessed with the method-comparison analysis [32], popularised by Bland-Altman [33], and the absolute percentage error (APE).

In the Bland-Altman analysis run in the current study, the x-axis reported the full test measures, i.e. the reference measures. On the y-axis was the difference between the measures from the reduced test measures and the reference measures.

The reduced test measures are considered a good approximation of the criterion standard if the Bland-Altman analysis shows no bias and the limits of agreement are sufficiently narrow: the absence of bias indicates that the new measures are accurate; the tight limits of agreement suggest that these are precise.

Acceptable limits must be defined a priori [34], and deciding if the limits of agreement are sufficiently tight depends on what the new measure is used for [35]. This indication is recommendable and relatively easy to implement for widely used measures such as gait speed and TTD. However, judging the maximum tolerable amplitude of the limits of agreement is more challenging for newer, less investigated measures, such as those from the inertial sensors.

For this reason, APE was also calculated as follows:

1 $$APE=100\times \left|\frac{N-R}{R}\right|$$

With $N$: new measure (here the measures from the reduced tests, i.e. Trial_2 − 3_ measures), $R$: reference measure (here the measures from the five repeat tests, i.e. the reference measures).

The APE 95th percentile (APE_95%_) was chosen as an agreement indicator.

#### SEM estimation with linear mixed-effects models

In the Classical Test Theory (CTT) framework, a single *measure* is the sum of the *true* quantity of the measured variable plus the *measurement error*. Under the assumption of (i) random, (ii) uncorrelated errors and (iii) errors uncorrelated with the true value of the measured variable, the variance of measures (i.e. the observed variance, in the CTT jargon) results from the sum of the true and error variances [4, 36].

The ANOVA is commonly used to disentangle the different sources of variation, with variance estimates derived from ANOVA mean square values [5, 37].

In particular, the total variance is modelled from the sum of the between-subjects mean square and the within-subjects mean square, with the within-subjects mean square equalling the error variance [5]. In this regard, SEM is simply the square root of the error variance [4].

As an alternative to ANOVA, linear mixed-effects models (LMMs) [38] can decompose the variance in different sources and isolate the measurement error variance [20].

LMMs are an invaluable statistical method for the analysis of complex datasets, such as those, common in medicine, made of contrasting groups of participants (e.g. patients vs. controls; patients receiving different treatments) assessed repeatedly in time (e.g. before and after treatments).

The formula of an LMM has three parts, one for handling fixed effects, another for random effects and a third consisting of an error term.

The fixed effects are used to represent sources of systematic variation in the data. In the regression jargon, the fixed effects correspond to the predictors.

Random effects account for variations within clusters, accounting in this way for an amount of variance of the response variable which is not explained by the fixed effects. For example, random effects can be used to model the variability of a cluster of non-independent measures, such as repeated data collected by an individual at a time point.

Finally, LMMs also contain an error term, which accounts for the residual variance not explained by the fixed or random effects.

Consider a clinical trial in which patients are randomized into two treatment groups and assessed three times. In each assessment session, multiple measures are collected from each participant, a common approach to address measurement error and obtain robust person estimates.

In the LMM framework the patient’s group (e.g. treatment A vs. treatment B), the assessment session (e.g. before, immediately after treatments and at follow-up) and the group × session are modelled as fixed effects. Random effects would be here the subjects, modelled as random intercepts and, in a maximal model [30], the assessment session, modelled as random slopes.

In this example, LMMs would be used for significance testing and an Analysis of Variance could be run on the LMM showing, say, that the response variable is significantly improved after treatment compared to before.

To put it simply, the fixed effect returns the average improvement in the response variable in the sample after treatments. The random intercept component specifies that each participant has a unique baseline value for the response variable and the random slope that each participant can improve to a different extent at the treatment end (i.e. that each participant has their slope of improvement).

In addition to significance testing, LMMs can also be used for prediction or, as done here and mentioned above, for variance decomposition to estimate the standard error of the measurement. This last application of LMMs is still unconventional and will be better developed in the following paragraphs.

Among the strengths of LMMs, in addition to the aforementioned ability to manage data not independent of each other, there is their robustness to missing data and the possibility they offer to easily control for confounding effects [37].

The participants recruited in the current study received physiotherapy and occupational therapy between the two assessment sessions. Indeed, they completed a rehabilitation program to improve their balance and gait. In line with our former findings [27, 28], it is reasonable to expect that, on average, the patient’s gait and balance at T_1_ were likely better than at T_0_. This fact violates the “steady-state assumption”, i.e., that the patient does not evolve, which is fundamental to SEM estimation in CTT [21].

In a typical CTT study in which a clinical measure’s reliability and SEM are calculated, subjects are measured twice, usually one or two weeks apart. This time interval between the two measurements is considered long enough to prevent recall but short enough not to change the “true” value of the measured variable [39]. In this scenario, it can be reasonably assumed that the true value of the variable remains the same in the two sessions. Any difference in measurement between the two sessions can then be attributed only to the measurement error.

LMMs provide a suitable solution if the steady-state assumption is not valid [21]. The idea is straightforward. Therapeutic exercise is expected to improve gait and balance in neurological patients, thus causing a change between T_0_ and T_1_ in the variables of interest and measures. By using LMMs, this change in time can be estimated and removed: the steady state is created mathematically.

In the current study, the following LMM model was used to estimate SEM of measurement Y (say Y is TTD, but the next holds for any of the mobility measures considered here):

2 $${Y}_{si}=({\beta }_{0}+{I}_{0i})+({\beta }_{1}+{I}_{1i}){X}_{s}+{r}_{si}$$

With ${Y}_{si}$, the predicted TTD for session $s$ and individual $i$;

${\beta }_{0}$, the TTD baseline level (i.e. the model’s intercept); the TTD at T_0_, when the predictor equals zero;

${I}_{0i}$, the random intercept component, indicating the deviation from ${\beta }_{0}$ for subject $i$;

${\beta }_{1}$, the session effect (i.e. the model’s slope); the change in TTD associated with a predictor’s change;

${I}_{1i}$, the random slopes component, indicating the deviation from ${\beta }_{1}$ for subject $i$;

${X}_{s}$, the predictor variable, here session, which takes values 0 for T_0_ and 1 for T_1_;

${r}_{si}$, the residuals, calculated for each $i$ in each session $s$.

The model also specifies that ${r}_{si}$, ${I}_{0i}$ and ${I}_{1i}$ are normally distributed, have mean equals zero, and each their variance. In particular, ${r}_{si}$ variance is labelled ${\sigma }^{2}$. Moreover, LMMs also consider the covariance between ${I}_{0i}$ and ${I}_{1i}$.

Including the random intercepts and slope terms allows the model to consider two clinically essential aspects.

First, it is unrealistic that all participants have the same TTD at baseline (i.e., that ${\beta }_{0}$ is the same for all participants). Instead, it seems more likely that at T_0,_ they are distributed around an average value.

Second, it is equally unrealistic for a participant to change the same after the rehabilitation (i.e., that ${\beta }_{1}$is the same for all). Even if unexpected, some individuals could worsen, for example, due to the occurrence of a complication. Moreover, even if all persons would improve, the amount of improvement (i.e. the responsiveness to rehabilitation) is likely to be different in different patients.

When LMMs are used for estimating SEM, it is assumed that deviations in the observed measure from the model’s prediction (i.e. the model’s residual) are caused by the measurement error. Hence:

3 $$SEM=\sqrt{{\sigma }^{2}}$$

And once SEM has been estimated, the 95% MDC can be calculated as customary:

4 $$95\% MDC=1.96\times \sqrt{2}\times SEM$$

It is worth stressing that, according to Eq. (2), the measured variable (in the example the TTD) depends, and only depends, on the assessment session (i.e. if it has been collected in T_0_ or T_1_) and the person being evaluated (and the estimated random effects variances and covariances and residuals variance). Any additional, unmodeled source of variation that moves the observations away from the model’s predictions and causes an increase in residuals is included in the residual variance.

The following will make this last point clear. In the current study, in each assessment session, two trials were repeated. It can be reasonably assumed that the quantity of the variable of interest, say TTD, does not change within a session (in a sense, a within-session steady-state assumption holds). Therefore, any difference in measures from Trial_2 − 3_ and Trial_4 − 5_ can be attributed to measurement error.

However, the LMM Eq. 2 does not explicitly model TTD as affected by the trial. In other words, the trial is not considered a source of TTD variation and any trial effect on TTD remains unquantified. Instead, any within-session difference in the measures from Trial_2 − 3_ and Trial_4 − 5_, a difference attributed to the measurement error, would be included in the residual variance of model 2.

#### Complying with the linear mixed-effects models’ assumptions

Equation (2) LMM relies on two assumptions: residuals are normally distributed and have constant variance. Therefore, when built on LMMs, SEM and MDC also rely on these assumptions.

Quantile-quantile plot and the absolute residuals plot as a function of the predicted values are used here to assess residuals’ normality and homoscedasticity. If these are violated, the response variable is ln-transformed. Notably, if the predictor is ln-transformed, the MDC obtained from the LMM is meant to be applied to ln-transformed measures.

Say that ω needs to be ln-transformed and that a patient improved their peak angular velocity during turning at rehabilitation end. This improvement is a real improvement if:

$$\text{ln}{{\omega }}_{{T}_{1}}- \text{ln}{{\omega }}_{{T}_{0}}>MDC$$

This inequality can be rearranged as follows:

$$\text{ln}\left(\frac{{{\omega }}_{{T}_{1}}}{{{\omega }}_{{T}_{0}}}\right)>MDC$$

$$\frac{{{\omega }}_{{T}_{1}}}{{{\omega }}_{{T}_{0}}}>{e}^{MDC }$$

$$\frac{{{\omega }}_{{T}_{1}}- {{\omega }}_{{T}_{0}}}{{{\omega }}_{{T}_{0}}}>{e}^{MDC }-1$$

Therefore, when the measure is ln-transformed, the MDC can be understood as the baseline measure’s minimum percentage change exceeding the measurement error.

#### Longitudinal agreement between the 95% MDCs and the “all five repetitions better than the best” method

We have previously proposed a method, referred to in the current study as “all five repetitions better than the best”, which is considered here the reference method to determine if a patient’s mobility measure has improved (or worsened) after rehabilitation [28]. According to this method, a participant improved if all five repetitions in the T_1_ session were better than the best repetition in T_0_. Conversely, if all five repetitions in the T_0_ session were better than the best repetition in T_1_, the participant got worse.

The accuracy of the 95% MDC was assessed by evaluating their agreement with the “all five repetitions better than the best” method. Since the agreement between these two methods is about the change of the patients in time, it will be referred to as a “longitudinal agreement” to avoid confusing it with the cross-sectional agreement assessed in the previous analyses.

Two indices have been used to assess longitudinal agreement: the weighted Cohen’s kappa and Youden’s J.

For Cohen’s kappa analysis, participants were classified as improved, stable and worsened according to the 95% MDC (Trial_2 − 3_ measures) and the reference method.

Cohen’s kappa is a chance-corrected agreement measure [40]. The weighted variant (squared weights) considers that disagreement can have different seriousness. For example, consider a patient flagged as improved by the reference methods but indicated as stable by the 95% MDC. If the 95% MDC had identified this patient as worsened, it would have been a more severe error.

While the intuition behind chance-corrected agreement methods is valuable, Cohen’s kappa can give paradoxical and unreliable results with some datasets. For this reason, it has been recommended that the results of a complementary analysis, essentially based on sensitivity and specificity, are presented alongside Cohen’s kappa [41]. Youden’s J [42] was calculated here for this purpose.

Participants were dichotomised into stable and changed (i.e. significantly got better or significantly got worse) according to the 95% MDC and the reference method. The number of true and false positives and negatives is counted (e.g. true positives: number of participants that changed their status according to the 95% MDC and the reference method), and Youden’s J is calculated.

Youden’s J ranges from 0 to 1 (with 1 meaning there are no false positives or negatives) and can be interpreted as the probability of correctly classifying a patient (i.e., correctly identifying a changed patient or a stable patient) penalised by the chance of making an error.

#### SEM estimation from reliability indices from the subset of steady participants: a control analysis

According to the previous analysis, participants can be divided into changed (i.e. participants who improved after rehabilitation or, less likely, who got worse) and stable (i.e. participants who did not change between sessions with respect to the variable of interest).

Patients who did not change represent a pool of individuals for whom the steady-state assumption holds. CTT reliability indices and the corresponding SEM can be calculated on this subset of participants and compared to the SEM returned by the LMM analysis.

For this complementary analysis, stable participants were defined according to the complete five repetitions tests (i.e., the reference tests), similarly to what was done in the longitudinal agreement analysis but applying stricter criteria.

A single participant was considered stable, i.e. the steady-state assumption was verified in them in the case of the following:

$$\left[\text{max}\left({T}_{0}\right)> \text{max}\left({T}_{1}\right) \right] \& \left[\text{min}\left({T}_{0}\right)< \text{min}\left({T}_{1}\right)\right]$$

With $\text{max}\left({T}_{0}\right)$, the maximum value of the variable on the five repetitions collected in session T_0_ (remaining symbols alike). Of course, the steady-state assumption also held if the opposite occurred.

In other words, stable participants are those for which the two extreme repetitions (e.g. the shortest and the longest TTD, the fastest and the lowest for gait speed) in a session (i.e. T_0_ or T_1_) are more extreme than the two extreme repetitions in the other session (i.e. T_1_ or T_0_).

Once the subset of unchanged participants was constituted, the Intraclass Correlation Coefficient (ICC) was calculated as the CTT reliability index.

A two-way mixed effect, absolute agreement (i.e. including systematic and variable error), single measurement model was used in this analysis as recommended for assessing test-retest reliability [43, 44]. Note that this model, nomenclature according to McGraw and Wong [18], corresponds to ICC_2,1_ of Shrout and Fleiss [19] from a strict computational point of view [5].

Only Trial_2 − 3_ measures from T_0_ and T_1_ have been considered for the ICC calculation.

To correspond with the primary analysis, ICC_2,1_ has been chosen over alternative models, such as ICC_2,k_.

As previously mentioned and as done elsewhere, e.g. [45]. , two metrics from two test repetitions were averaged to provide a single measure in this study. This one measure was used then as the response variable in the LMMs for the primary analysis. Hence, these measures were also included in the ICC models in place of the original metrics. Since a *single measure* was obtained from two metrics, a *single-measurement*, absolute agreement ICC model was chosen.

About the choice of ICC_2,1_, it is also worth noting that the mobility measures used here are, in a way, composite measures precisely because they are derived from two preliminary metrics. In this scenario, computing the ICC_2,1_ is similar to calculating the ICC_2,1_ of questionnaire total scores, where the questionnaire total score is the one measure that is derived from many metrics (i.e. the questionnaire items).

It should also be stressed that the subset size can differ for the different measurements and is the smallest for the measure with the highest responsiveness to rehabilitation [27, 28].

A sums of squares approach (i.e. ANOVA) was used for reliability estimation rather than regression (i.e. LMMs) to stay more in line with the CTT framework.

After estimating reliability, the SEM was calculated according to:

5 $$SEM={SD}_{{T}_{0}}\times \sqrt{1-ICC}$$

With ${SD}_{{T}_{0}}$, the standard deviation of the T_0_ measures.

Finally, the 95% MDC was obtained per Eq. (4).

All analyses were run in R version 4.2.3 “Shortstop Beagle” (The R Foundation for Statistical Computing). The library “lme4” [46] was used for fitting LMMs. Type 1 error probability was set at 0.05 and corrected in post hoc tests, according to Holm [47]. As done for the SEM estimation, when used for hypotheses testing, LMMs assumptions were assessed, and the response variable was ln-transformed if needed.

## Results

The participants’ sample consisted of 109 neurological patients, 43 females (39.5%).

Thirty-nine individuals were older than 80 (35.8%), and the participants’ median age was 78.4 years (1st to 3rd quartile = 72.3 to 81.5 years).

Sixty-six patients (60.6%) had a hemiparetic gait impairment secondary to a stroke, 23 (21.1%) had peripheral neuropathy of the lower limbs, and 20 (18.4%) had parkinsonism.

The median length of stay in rehabilitation was 35.0 days (1st to 3rd quartile = 24.0 to 47.0 days).

Overall, before rehabilitation (i.e. at T_0_), the participants suffered a moderate mobility impairment as indicated by their median gait speed (0.87 m/s; 1st to 3rd quartile = 0.70 to 1.11 m/s) and TTD (14.5 s; 1st to 3rd quartile = 11.6 to 18.8 s; reference tests).

### Agreement between the reduced and the reference mobility tests

**Fig. 2:**
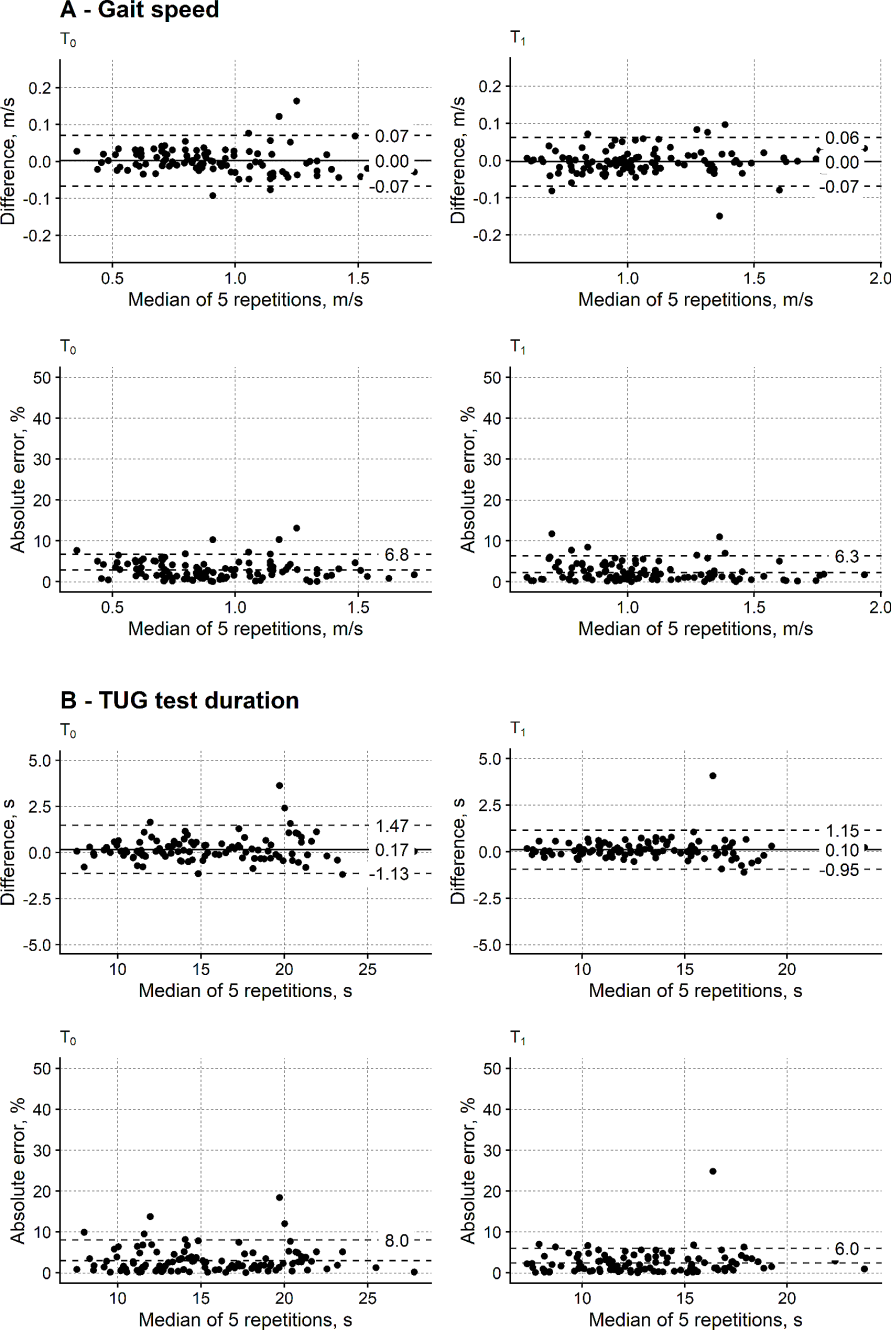
Cross-sectional agreement between the measures from the shortened gait and TUG tests and the reference variant. (**A**) agreement for gait speed; (**B**) agreement for TUG test duration (TTD). Agreement is reported separately for the first (T_0_, left) and the second (T_1_, right) assessment session. Upper plots in panels A and B are Bland-Altman plots of the difference between measures from the shortened and the reference test (y-axis) as a function of the reference test measures (x-axis). Reference test: the test is repeated five times, metrics collected from each repetition and the person’s measure is the median of five metrics. Comparison (new, shortened) test: the test is repeated three times, the first repetition is discarded, metrics are collected from the second and third repetition, and the person’s measure is the mean of two metrics (i.e. Trial_2 − 3_ measures). Continuous horizontal line: bias (i.e. mean difference between measures); horizontal dashed lines: limits of agreement. Lower plots in panels A and B show the absolute percentage error (APE, y-axis) of the measures from the reduced tests as a function of the measures from the reference tests (x-axis). Lower horizontal dashed line: mean APE (label not shown for graphical reasons). Upper horizontal dashed line: APE 95th percentile. Each dot marks a person’s measure

Figure 2 shows the Bland-Altman plots and the APE for the gait speed and the TTD measured with the reduced and the five-repeat tests.

Regarding the gait speed (Fig. 2A), the bias from the Bland-Altman analysis is negligible (0.00 m/s) in both assessment sessions. The precision of the measures from the reduced walking test is also satisfactory, as indicated by the limits of agreement, which are reasonably narrow (0.07 m/s at most) at T_0_ and T_1_. No trend is apparent between the measures difference (y-axis) and the reference measures (x-axis), and no heteroscedasticity is found.

The APE analysis confirms the excellent agreement between the gait speed measured with the two tests. The mean APE was 2.8% and 2.3% in the first and second assessment session, respectively, and APE_95%_ is well below 10% (T_0_: 6.8%; T_1_: 6.3%).

Regarding the TTD (Fig. 2B), bias on T_0_ (0.17 s) and T_1_ (0.10 s) is slight, and the amplitude of the limits of agreement is satisfactory (T_0_: 1.30 s; T_1_: 1.05 s).

The study of the APEs confirmed these findings of the Bland-Altman analysis. The mean APE from the T_0_ assessment is 3.0%, and 2.4% from T_1_. Similarly to gait speed, the APE_95%_ is smaller than 10% on T_0_ (8.0%) and T_1_ (6.0%).

**Fig. 3:**
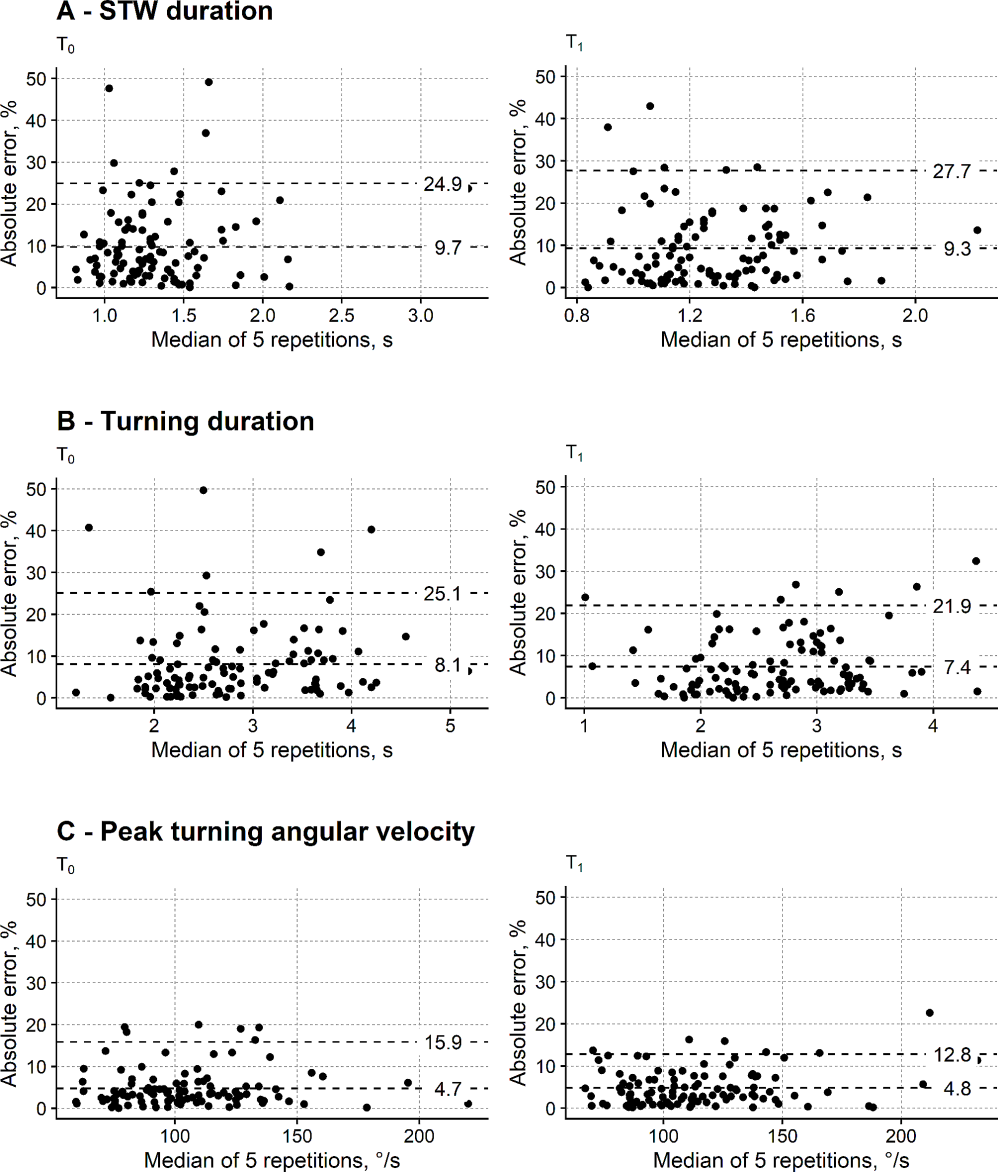
Cross-sectional agreement between the measures from the shortened and the reference TUG test: measures from the inertial sensors. Absolute percentage error is depicted. Same graphics as Fig. 1. STW: sit to walk

Figure 3 shows the agreement between the reduced and the full TUG test for the STW duration, turning duration and ω.

The APE_95%_ of the STW and the turning duration are the most prominent (> 20%) in both assessment sessions. A better APE_95%_ was found for ω (T_0_: 15.9%; T_1_: 12.8%), even if larger than the APE_95%_ of the gait speed and TTD.

### Within- and between-sessions time course of the measurements from the mobility tests

**Fig. 4:**
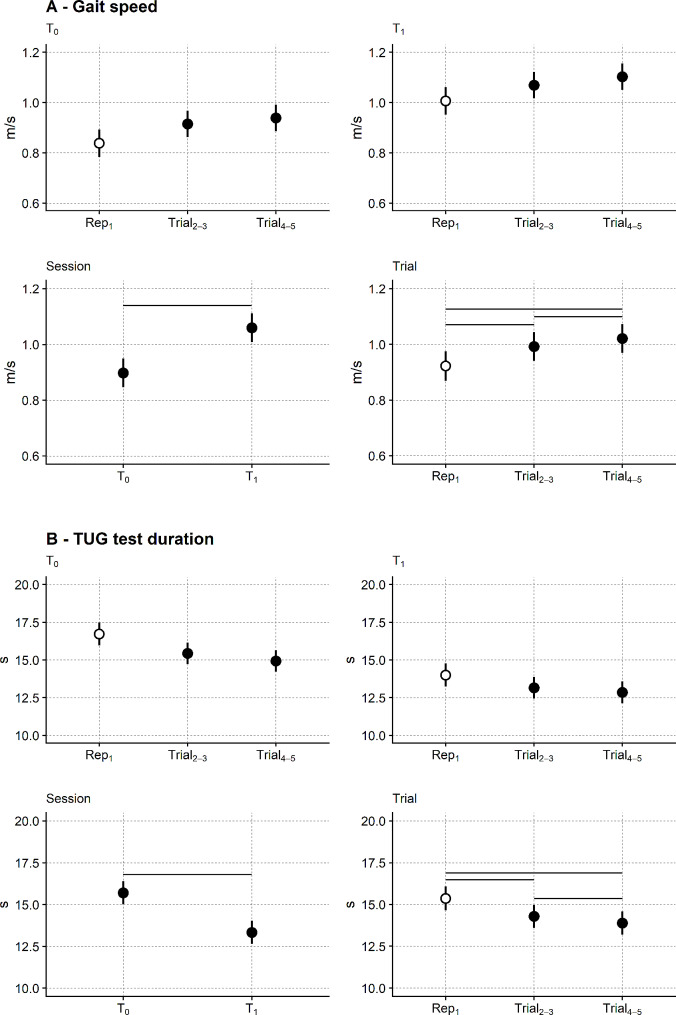
Time course within and between sessions of gait speed and TUG test duration. Gait speed (**A**) and TUG test duration (**B**) from the reduced tests. White dots: metric from the first test repetition, i.e. the habituation repetition. Black dots: measures from Trial_2 − 3_ and Trial_4 − 5_. Lower graphs in panels A and B report the significance analysis from ANOVA on linear mixed-effects models. Session (left) and trial (right) predictors were significant, while their interaction was not both for the gait speed and the TUG test duration. Horizontal bars mark significant contrasts on post hoc testing. Estimated marginal means and their 95% confidence interval from linear mixed-effects models are depicted. TUG test duration was ln-transformed to comply with regression assumptions. It is shown here as untransformed for graphical reasons

Figure 4 illustrates the gait speed and the TTD time course in the two sessions (i.e. T_0_ and T_1_). Measures from the two trials (Trial_2 − 3_ and Trial_4 − 5_) are shown together with the first habituation repetition.

In both sessions, participants’ performance was poorer in the first repetition than in the subsequent trials: gait speed was lower, and the total TUG test duration increased. Participants moved faster at T_1_, i.e., after the end of the rehabilitation, than at baseline, i.e. T_0_.

The ANOVA substantiates these findings. Session (F_1,979_ = 482.5; *p* < 0.001) and trial (F_2,979_ = 52.8; *p* < 0.001) were significant gait speed predictors, while their interaction was not (F_2, 979_ = 0.33; *p* = 0.718).

In particular, gait speed was significantly higher on T_1_ (1.06 m/s, 95%CI: 1.01 to 1.11 m/s) than on T_0_ (0.90 m/s, 95%CI: 0.85 to 0.95 m/s).

According to posthoc testing, regardless of the session, gait speed was significantly lower in the first repetition (0.92 m/s, 95%CI: 0.87 to 0.98 m/s) than in Trial_2 − 3_ (0.99 m/s, 95%CI: 0.94 to 1.04 m/s; *p* < 0.001) and Trial_4 − 5_ (1.02 m/s, 95%CI: 0.97 to 1.07 m/s; *p* < 0.001). Despite the few centimetres per second, gait speed was higher on Trial_4 − 5_ than Trial_2 − 3_ (*p* < 0.001).

Regarding the total TUG test duration, the ANOVA again showed the significance of session (F_1, 981_ = 382.9; *p* < 0.001) and trial (F_2,981_ = 43.9; *p* < 0.001) factors, while their interaction did not reach the significance level (F_2,981_ = 0.66; *p* = 0.518).

Similarly to gait speed, the total duration of the TUG test significantly improved (i.e. decreased) in T_1_ (13.3 s, 95%CI: 12.6 to 14.0 s) as compared to T_0_ (15.7 s, 95%CI: 15.0 to 16.4 s).

Post hoc comparisons showed that, in both sessions, the total TUG test duration was significantly longer in the first repetition (15.4 s, 95%CI: 14.6 to 16.1 s) than in Trial_2 − 3_ (14.3 s, 95%CI: 13.6 to 15.0 s; *p* < 0.001) and Trial_4 − 5_ (13.9 s, 95%CI: 13.2 to 14.6 s; *p* < 0.001). The difference between Trial_2 − 3_ and Trial_4 − 5_, although tiny, was also significant (*p* = 0.003).

**Fig. 5:**
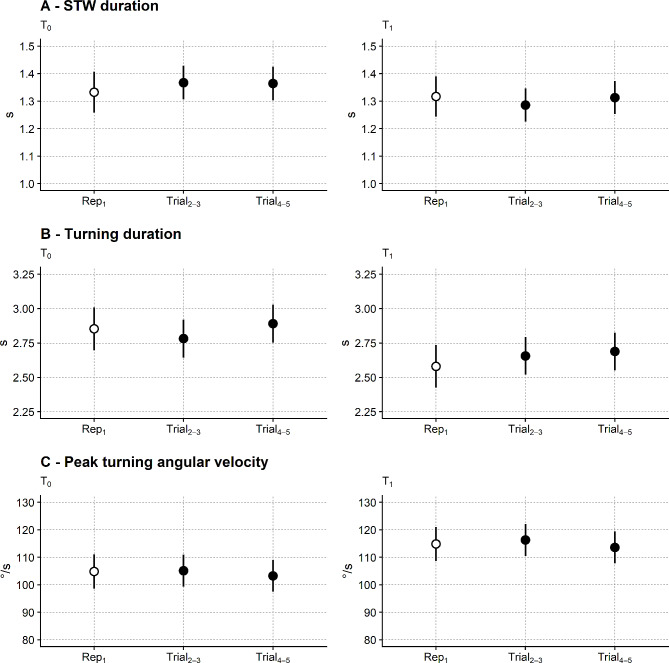
Time course of the TUG test measurements from inertial sensors. Same graphics as Fig. 3. STW: sit to walk. The main finding from statistical testing (ANOVA) was a significant difference between the two sessions. All three variables were ln-transformed and reported here as untransformed

About the TUG test measures from inertial sensors (time course provided in Fig. 5), STW and turning duration and ω showed a similar pattern of significance on ANOVA.

STW duration was significantly lower at T_1_ (1.31 s, 95%CI: 1.25 to 1.36 s) than at T_0_ (1.35 s, 95%CI: 1.30 to 1.41 s; F_1,956_ = 4.8; *p* = 0.023). Turning duration was also significantly lower in T_1_ (2.64 s, 95%CI: 2.52 to 2.77 s) than T_0_ (2.84 s, 95%CI: 2.72 to 2.97 s; F_1,952_ = 27.0; *p* < 0.001) while ω_peak_ significantly increased in T_1_ (115 °/s, 95%CI: 109.4 to 120 °/s) compared to T_0_ (104 °/s, 95%CI: 98.9 to 110.0 °/s; F_1,950_ = 111.5; *p* < 0.001).

No trial effect was found for STW duration and ω, while a tiny trial effect was found for the turning duration. The session times trial interaction was not significant in the three models.

The complete details of the statistical analysis are provided in Supplementary Materials 1.

### SEM and 95% MDC of the mobility tests measurements

The SEM and the 95% MDC of the gait and TUG test measurements from the reduced tests are provided in Table 1.

**Table 1:** 95% MDC of gait and TUG tests mobility measures

	Complete sample LMMs			Steady participants CTT		
	SEM	MDC	e^MDC^	SEM	MDC	e^MDC^
gait speed, m/s	0.047	0.130	*1.139*	0.055	0.152	*1.167*
TUG test duration (ln), s	0.052	0.143	1.154	0.058	0.159	1.173
STW duration (ln), s	0.152	0.422	1.524	0.169	0.469	1.599
Turning duration (ln), s	0.123	0.340	1.405	0.135	0.374	1.453
ω (ln), °/s	0.080	0.222	1.249	0.093	0.257	1.294

LMMs: linear mixed-effects models; CTT: classical test theory; STW: sit to walk; ω: peak angular velocity along the vertical axis during the first turning phase of the TUG test; SEM: standard error of the measurement; MDC: 95% minimal detectable change; e: Euler’s number. e^MDC^ on the gait speed row (italics) is from LMMs or ANOVA with ln-transformed gait speed as the predictor. Complete sample LMMs: SEM and MDC were calculated from the full participants’ sample using random intercepts and slopes LMMs with the session (T_0_ vs. T_1_) as a fixed effect. Steady participants CTT: SEM and MDC were calculated from the subset of stable participants (i.e. strictly unchanged after rehabilitation) using ANOVA.

All the measurements were ln-transformed to better comply with the assumptions of the regression models used for SEM estimation. Gait speed was the only of the five measures acceptably performing when inputted untransformed in regression.

Similarly to the previous agreement analysis, STW and turning duration worked poorly. In fact, from the 95% MDC value calculated on the ln-transformed data, the STW duration should decrease (or increase) by about 50% of the baseline value to conclude a real change. Similarly, the turning duration would have to change by about 40% from the baseline value.

The smallest 95% MDCs were those of the TTD (15% of the baseline measurement needed to conclude a real TTD change) and that of the gait speed (14% change).

Between the two extremes, the 95% MDCs of the ω was about 25%.

### Longitudinal agreement between the 95% MDCs from the reduced and the reference tests

According to the 95% MDC of Trial_2 − 3_ measures, gait speed and TTD had the highest responsiveness (Table 2). In fact, according to these measurements, more than half of the participants significantly improved at the end of the rehabilitation.

**Table 2:** Longitudinal agreement between the 95% MDC and the reference method

	Reference			95% MDC		
	got better	unchanged	got worse	got better	unchanged	got worse
gait speed (%)	53 (48.6)	51 (46.8)	5 (4.6)	57 (52.3)	47 (43.1)	5 (4.6)
TUG test duration – ln (%)	49 (45.0)	55 (50.5)	5 (4.6)	57 (52.3)	46 (42.2)	6 (5.5)
STW duration – ln (%)	12 (11.0)	95 (87.2)	2 (1.8)	8 (7.3)	97 (89.0)	4 (3.7)
Turning duration – ln (%)	12 (11.0)	94 (86.2)	3 (2.8)	10 (9.2)	88 (80.7)	11 (10.1)
ω – ln (%)	23 (21.1)	84 (77.1)	2 (1.8)	24 (22)	75 (68.8)	10 (9.2)

STW: sit to walk; ω: peak angular velocity along the vertical axis during the first turning phase of the TUG test. The number of subjects and their percentage (between brackets) is reported. Reference: reference method (i.e. the “all five repetitions better than the best” method) for establishing the patient’s change after rehabilitation (i.e. in session T_1_ compared with T_0_). 95% MDC: patient’s change is defined according to the 95% minimal detectable change (MDC) from the reduced tests. After rehabilitation, participants got better (i.e. increased their gait speed, decreased their TUG test, STW and turning duration or increased ω), did not change (unchanged) or got worse

STW and turning duration had the lowest responsiveness since about 90% and 80% of participants did not change above the 95% MDC.

Again, ω results were in between the gait speed and TTD on the one hand and those of the STW and turning duration on the other, with about 20% of participants improving after rehabilitation per the ω 95% MDC.

Table 2 also reports the responsiveness analysis for the reference method.

**Fig. 6:**
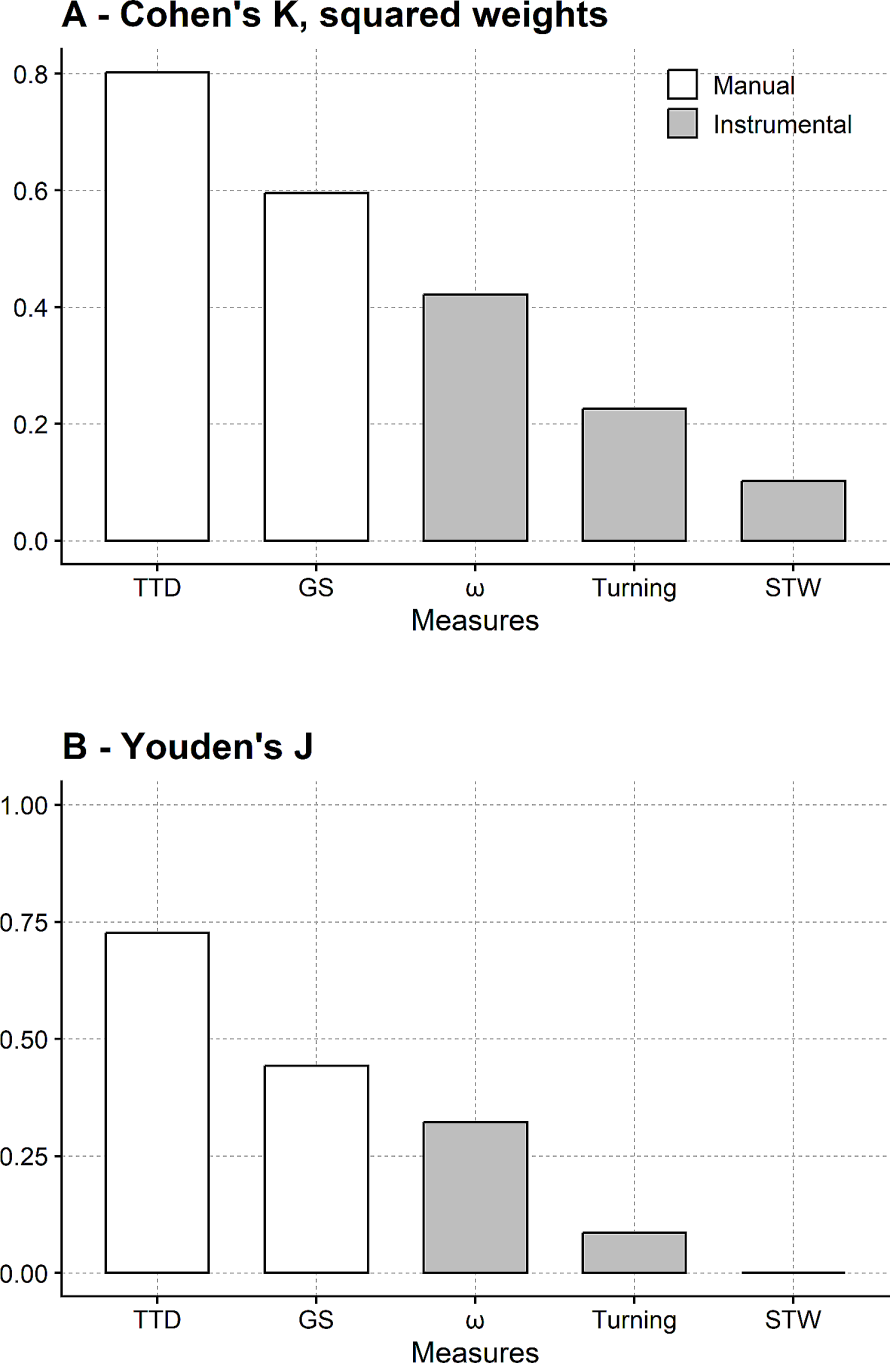
Longitudinal agreement between the 95% MDCs from the reduced tests and the reference method for assessing the patient’s change in time. Reference method: “all five repetitions better than the best” method. Novel method: 95% MDC from linear mixed-effects models run on Trial_2 − 3_ and Trial_3 − 4_ data from T_0_ and T_1_ sessions. (**A**) participants are classified as unimproved, improved or worsened, and Cohen’s K (squared weights) is used as the agreement index. (**B**) participants are classified as stable or changed (i.e. worsened or improved), and Youden’s J is calculated as the agreement index. Measures are ranked according to their agreement with the reference method (the higher the index, the better the agreement). Grey bars, i.e. instrumental measures, are from the TUG instrumented with inertial sensors. White bars, i.e. manual measures, are from a stopwatch. TTD: TUG test duration; GS: gait speed; ω: peak vertical angular velocity during the first turning phase; Turning: duration of the first turning phase; STW: sit-to-walk duration. Youden’s J of STW was negative (-0.04) and plotted as zero for graphical reasons

The agreement between the 95% MDC and the reference methods is displayed in Fig. 6.

The weighted Cohen’s kappa (Fig. 6A) indicated substantial agreement between the two methods for TTD and moderate agreement for gait speed and ω. The agreement for turning duration was fair, while that of STW duration was poor.

This ranking is confirmed when the longitudinal agreement was assessed with Youden’s J (Fig. 6B).

### 95% MDCs from the subset of steady participants: a control analysis

The gait speed of ten participants was strictly unchanged (*p* = 0.846, Wilcoxon signed rank exact test), while TTD was strictly unchanged in 18 participants (*p* = 0.966). As expected, given the analysis above, the number of participants strictly unchanged was larger for the STW and turning duration (32 for both, *p* = 0.106 and *p* = 0.065, respectively). No modification of ω was found in 24 individuals (*p* = 0.114).

The SEM and the 95% MDC calculated on the sample of steady patients are reported in Table 1.

The MDC derived from the ICC is larger than that from LMMs for all five mobility measures. However, this difference is quite small, the smallest for TTD and gait speed and the largest for STW duration.

For example, according to the 95% MDC from LMMs, TTD should decrease by about 15% to conclude a significant change in patient’s mobility. According to the 95% MDC from the steady state patients, the TTD reduction should be about 17%.

## Discussion

The current study reports the SEM and the 95% MDC of the gait speed and TUG test duration, measured with a stopwatch, and measures from the sit-to-walk and turning phase of the TUG test, recorded with an inertial measurement unit.

The 95% MDC of the gait speed and the TTD were the smallest, while those of the STW and turning duration were the largest. The 95% MDC of ω was between these extremes. More precisely, gait speed and TTD should change by about 15% of their baseline value to conclude an actual patient’s modification. This change is 25% for ω, but 40% or more for STW and turning duration.

This study is certainly not the first in which the SEM and the 95% MDC of gait and TUG test measures have been calculated, but our work nonetheless offers an important addition to the current literature.

Firstly, to our knowledge, this is the first time LMMs have been used to estimate SEM of gait and mobility measures from data collected from neurological patients undergoing rehabilitation due to their balance impairment. This represents an important methodological novelty in the field of mobility assessment.

Second, further investigation of the SEM and the MDC of gait and other mobility measures is still needed. Indeed, while many previous studies report the reliability of gait and mobility measures, the number of studies reporting the MDC of these measures is rather limited (e.g. [16]. ). A systematic review [14] concluded that the MDC is the least frequently assessed psychometric property of walking and mobility measures in neurological patients. Systematic reviews also indicated the need for further investigations of the SEM and MDC of gait and mobility measures in different diseases, including neurological ones [16]. It should also be noted that the SEM and MDC of the mobility measures obtained with inertial measurement units are probably the least known.

### Estimating the Standard Error of the Measurement when the steady-state assumption does not hold: what witchcraft is this?

Patients are assessed twice in a typical test-retest experiment for estimating SEM or the reliability of mobility measures, usually one week (e.g. [48]). or a couple of weeks apart (e.g. [49]).

In this period, patients are appropriately instructed to not to exercise or participate in rehabilitation, and a few weeks is too short for a disease to progress. Therefore, it can be assumed that there is no systematic changes in the effective amount of the variable under examination to occur during the test-retest interval. As a result, if this steady-state assumption is complied with, any measurement change between the two assessment sessions can be attributed solely to the measurement error.

The steady-state assumption is central to SEM estimation in the CTT framework [21]. More technically, when SEM is estimated, CTT assumes “parallel tests”, namely that measures have the same “true” value and error variances in the two assessment sessions [50].

In the current study, the steady-state assumption is blatantly violated because of a treatment effect. In cases such as this, the LMMs represent a valuable solution for estimating reliability and SEM since they can easily account for changes in measurement over time. According to some Authors [21], LMMs actually make the steady-state assumption, i.e. that the quantity of the measured variable does not change between the test and retest sessions, unnecessary.

Regarding the steady-state assumption, it is worth mentioning that for some of the literature this assumption is “extreme”, “unrealistic in many practical situations”, and “often violated in practice” [21, 50].

To our knowledge, this is the first study in which data before and after rehabilitation are entered into LMMs to estimate the SEM of gait and TUG test measures.

While this approach is new when applied to the study of mobility measures, it has been used in other fields. For example, reliability indices (which provide for SEM estimation) have been calculated by applying LMMs to data from clinical trials, which, as in the case dealt with here, are unstable because of a treatment or time effect [20]. In this case, similar to what we did here, LMMs have been used to account for time and treatment effects by entering them as fixed effects of the model [20].

The major limitation of the method used here for SEM estimation in which the treatment effect is removed thanks to LMMs is that any between-session difference in measures genuinely caused by measurement error is also removed (in a sense, the time effect is “taken out” from data, not just the treatment). Three points should be discussed about this.

First, two SEM types have been defined: SEM consistency and SEM agreement, the former considering only the residual variance and the latter the residual variance plus variance due to systematic differences (e.g. between raters, between sessions) [17, 39]. If no bias is present, consistency and agreement coincide.

When time is included as a fixed effect in the LMMs for SEM estimation, the systematic difference between sessions is removed, and SEM consistency is obtained.

Even if some Authors have recommended SEM agreement [39], SEM consistency is also used alongside SEM agreement. For example, the MDC calculated as the limits of agreement from the Bland-Altman method applied to a test-retest, steady-state study is an MDC consistency [17, 39, 51]. Some Authors consider the MDC, as well as the limits of agreement, to be equivalent indicators of measurement errors (e.g. [15]). Moreover, according to the Risk of Bias checklist for systematic reviews of Patient Reported Outcome Measures developed by the COnsensus-based Standards for the selection of health Measurement INstruments (COSMIN) [52], studies reporting the SEM, the MDC, or the limits of agreement should be considered “very good”. Studies in which only the limits of agreement are calculated are still considered as “adequate”.

Second, it is important to note that studies employing conventional steady-state, test-retest experimental designs pointed out the absence of bias for mobility measures between the two assessment sessions. For example, no systematic difference has been found for several gait variables in walking tests in chronic stroke patients [48]. A bias is not explicitly underlined in a systematic review of the reliability of gait measures [53].

Finally, we run a control analysis to experimentally investigate the differences between the SEM returned by LMMs with treatment as the fixed effect and SEM from test-retest steady-state reliability indices. For this analysis, only a subset of participants who remained strictly stable (i.e. neither improved nor worsened) after the rehabilitation was considered in order to be able to calculate steady-state test-retest reliability and SEM. Even if the SEM calculated with this strategy was larger than that from the LMMs for all five mobility measures, the difference between the two indices was small and likely of little practical significance.

The larger SEM could be because an agreement and a consistency SEM have been calculated with the reliability and the LMMs analyses, respectively. However, it can also be speculated that a (slightly) larger measurement error could have happened idiosyncratically in the strictly unchanged patient subset, which remained stable precisely because of this inflated measurement error.

### Are the reduced tests good enough?

In all our previous works, the median metric from the five repetitions of the gait and TUG tests was used as the patient’s mobility measure (e.g. [12, 27–29]). These studies aimed to assess the validity and responsiveness of mobility measures, mainly from the TUG test, as balance measures, i.e., a person’s ability not to fall.

As explained in the methods section, measures from reduced, shortened tests were used in the current work to specify maximal LMMs [30] and estimate SEM more accurately.

The cross-sectional (and longitudinal) agreement between the measures from the novel, shortened test versions and our reference was assessed as a preliminary, in a sense mandatory, step so that our previous findings (e.g., that measures from the turning TUG test phase are valid balance measures) can also be applied to the new measures.

Even if this control analysis was run to give continuity to our previous studies, and statistical reasons prompted the development of a shortened version of the mobility tests, its findings are also attractive for another reason.

To our knowledge, there is no agreement on the number of times the walking and the TUG test should be repeated. For example, in some studies, these tests are performed just once in an assessment session [54], while in other studies, the tests are repeated even ten times per session [53].

A two repeats test is clearly more readily applicable than a longer one, and the availability of a more straightforward test is vital in the clinic and research setting. In our experience, repeating the walking and TUG tests five times can be somewhat burdensome. This fact is especially true if additional measures are collected in an assessment session, such as balance scales or questionnaires for assessing the self-perceived balance or the 6 min walking test, which is often the case. Evaluating if a shortened test returns measurements as good as those from a longer one seems thus valuable.

We show that cross-sectional and longitudinal agreement is satisfactory for the gait speed and TTD measured with a simple stopwatch. The two agreement types are poor for the STW and turning duration, while those of ω are acceptable.

These conclusions are about the agreement of measures of single persons (i.e. measures precision) and are sustained by the 95% percentile APE values. It should be noted that when sample mean measures are considered, the mean APE points out a reasonable agreement (i.e. good accuracy) for all the measurements evaluated here.

The main message is that when interested in measuring a single person, for example, estimating their risk of falling or change after treatments, the “repeat three times, leave the first out and average the remaining two” assessment variant returns gait speed and TTD measures with high validity. The validity of ω, a balance measure, from this method is also acceptable.

### Are the 95% MDCs calculated here in line with those of previous studies?

We already showed that the MDCs calculated on before-after rehabilitation data with LMMs align with those obtained estimating reliability from a steady-state, test-retest experiment (see above). Moreover, our findings are in line with those of previous investigations.

In a systematic review of old persons with dementia [55], the 95% MDC of the gait speed measured with the 10 m walking test was 0.16–0.17 m/s.

In another systematic review [14], the MDC of the gait speed measured with the 10 m walking test was 0.15 m/s in stroke patients when walking at their usual pace. A slightly higher value (0.19 m/s) was found in the same study in Parkinson’s disease.

A systematic review assessing the reliability and the MDC of gait parameters measured with instrumented walkways [53] showed that 95% MDC of gait speed was about 0.13 m/s when different conditions, including neurological disease, were considered altogether. The largest 95% MDC was found in multiple sclerosis (0.28 m/s) and the smallest in stroke and old persons (about 0.11 m/s).

For a measure to be acceptable, its MDC must be smaller than the minimal clinically important difference (MCID) [52]. Otherwise, the measurement error is so significant that the measure cannot index changes in patients deemed clinically significant.

A recent study [49] calculated the MDC and the MCID of comfortable walking speed in stroke patients, the former being 0.13 m/s and the latter 0.18–0.25 m/s. Notably, the gait speed MDC that was found was comparable to the MDC found by [49] and, most importantly, lower than their MCID.

Comparing the MDC of the TTD is less straightforward since it is often reported as seconds rather than percentages, as we did. For example, the TTD 95% MDC in dementia ranged from 2.4 to 7.7 s [55].This fact leads us to point out an issue rarely considered in studies where the reliability or SEM are calculated. Both when SEM is estimated from ICC and ANOVA or using LMMs, SEM relies on these models’ assumptions, normality and homoscedasticity of residuals in the first place. For example, when variability increases as the measured values increase, as is always the case for TTD (e.g. [56]). , the data are heteroscedastic [57]. The assumptions of the models used for SEM estimation are violated: SEM estimation may therefore be unreliable.

Here, we show that the ln-transformation solves or improves adherence with the regression assumptions (Supplementary Materials 1). Therefore, it is advisable to express the MDC as a percentage change of the baseline measure.

Previous studies suggested that the 95% MDC can be considered acceptable if smaller than 30% of the mean test-retest measure [53].

Regarding the measures from the TUG with instrumental sensors, the STW and turning duration are beyond this threshold (while ω is below). Given the results of the cross-sectional agreement analysis, this finding is unsurprising.

Measurement error seems the largest for STW and turning duration. When the number of repetitions is lowered, the measurement error is less likely to be cancelled from trial to trial. A larger measurement error eventually inflates the 95% MDC.

The reason for a larger measurement error could be in these measures’ lower signal-to-noise ratio. For example, it is relatively simple to distinguish the TUG test end to that TTD can be obtained manually with a stopwatch. On the contrary, identifying the onset or the offset of the STW and turning phase is more challenging (e.g. signals do not start or do not return to zero).

### Study limitations and future developments

To our knowledge, it is the first time “unstable” data, in the psychometric sense, are used to assess the SEM of mobility measures. For this reason, we tested the simplest LMMs that allow estimating SEM, hence the current analysis should be considered introductory. Statistical packages are available, such as the R libraries “CorrMixed” [58] and “rptR” [59], which allow not only the variance partitioning which was run here but also provide additional tools for advanced analysis (e.g. confidence intervals of the estimates).

For example, we modelled the assessment sessions as before (T_0_) and after rehabilitation (T_1_) without considering the exact days (i.e. the length of stay in rehabilitation) between the two assessments. Future studies could also consider if the SEM changes as the length of stay increases as reliability may decrease with increasing time between test-retest sessions (e.g., learning effects may vanish).

In this study, in most instances, the response variable of the LMMs has been ln-transformed (e.g. TTD), primarily to adhere to the model’s assumption of residual normality.

Instead of transforming the response variable and computing the LMMs on this new variable, an alternate approach would have been to use more sophisticated formulations of the mixed models, such as the generalised LMMs [38].

For the reasons listed below, nevertheless, we chose not to pursue this alternative.

When mixed models are utilised for prediction or significance testing, using the generalised LMMs rather than the LMMs on the transformed response variable appears to be a suitable solution. Notably, some scholars could even think that this is a better option in this scenario.

However, utilising the generalised LMMs presents some difficulties when interested in variance decomposition to estimate the residual variance and SEM. Because of the link function, if the generalised LMMs were employed in this situation, the residual variance (and SEM eventually) would not be on the same scale as the response variable (i.e. the measure of interest). Conversely, using the LMMs and maintaining the SEM on the same scale as the (transformed) response variable makes more straightforward the application of the MDC in future research and clinical settings.

Furthermore, as the Methods section *“Complying with the linear mixed-effects models’ assumptions”* explains, when the ln-transformation is applied to the response variable, ln-transformed variables find an immediate practical meaning, even though technically the transformed variable is something different from the original (the two variables are monotonically related).

Lastly, it is important to emphasise again how unique this method is for SEM estimation. At this stage, we believe it is better to use simpler modelling techniques (e.g. LMMs) as opposed to more complex ones (e.g. generalized LMMs).

Only two sessions – before (T_0_) and after (T_1_) rehabilitation – were used to gather data for this investigation. However, it is worth noting that LMMs would allow estimating the SEM with no difficulty also in the case data were available from multiple sessions (e.g. before and after treatments and from early and late follow-up sessions) [20]. In this case, having more data available for statistical modelling would probably result in estimates (the SEM in the first place) that are more robust, indeed.

This study is preliminary also because different neurological syndromes have been grouped together, although united by the fact of being they are older persons with neurological pathologies with a high risk of falling. An obvious development would be comparing the SEM in the different diseases to assess if the SEM is the same for different diagnoses. Proving the absence of interaction between error and neurological disease would ease SEM applicability.

SEM of STW and turning duration should also be further assessed. The SEM of the measures from the five repeats test (i.e., the median of the five metrics) could be calculated as a starting point. As previously discussed, more repetitions are expected to lower the measurement error.

## Conclusions

Linear mixed-effects models provide valid estimates of test-retest SEM of mobility measures when the steady-state assumption does not hold, such as when mobility data are collected before and after rehabilitation.

The SEM and the 95% MDC of the gait speed, TUG test duration, and the vertical angular velocity peak in the TUG test’s first turning phase (ω, a balance measure) are small enough to be meaningfully applied in clinical and research settings.

These indices, calibrated here in neurological old persons, allow assessing the gait and balance modification in time in this population characterised by an increased falling risk.

### Electronic supplementary material

Below is the link to the electronic supplementary material.

Supplementary Material 1

## Data Availability

The dataset supporting the study’s main conclusions will be available on Zenodo upon manuscript acceptance and publication.

## Footnotes

1 In the current paper, we will call “metric” the measure of the variable of interest from a single test repetition. Metrics are in effect measures. However, to avoid confusion between measures of test repetitions and the subject’s measure (i.e. the subject’s gait speed and the like) we will use “measure” and “measurement” specifically to indicate the subject’s measure, which, as explained later, is returned by the median or the mean of several metrics.

## Abbreviations

ANOVA: Analysis of Variance
APE: Absolute percentage error
CI: Confidence interval
CTT: Classical Test Theory
ICC: Intraclass correlation coefficient
LMMs: Linear mixed-effects models
MDC: Minimal Detectable Change
SEM: Standard Error of the Measurement
STW duration: Sit-to-walk duration
T_0_: First assessment session
T_1_: Second assessment session
Trial_2 − 3_: Repetitions 2 and 3
Trial_4 − 5_: Repetitions 4 and 5
TTD: TUG test duration
TUG test: Timed Up and Go test
ω: Peak angular velocity along the vertical axis during turning
