# Supplementary material for: Minimal detectable change of gait and balance measures in older neurological patients: estimating the standard error of the measurement from before-after rehabilitation data thanks to the linear mixed-effects models
Source: J Neuroeng Rehabil. 2024 Apr 2;21:44. doi: 10.1186/s12984-024-01339-4 (PMC10986034; doi:10.1186/s12984-024-01339-4)
Supplement: Supplementary file 1 — Supplementary Material 1 [file 12984_2024_1339_MOESM1_ESM.docx]

**SUPPLEMENTARY MATERIALS 1**

This appendix reports the complete linear mixed-models (LMMs) analysis results. LMMs have been used for hypotheses testing and estimating the standard error of the measurement (SEM) of five mobility measures.

A particular emphasis is put on checking if the assumptions of normality and homoscedasticity of model residuals are fulfilled. For this reason, in most cases, the response variable has been ln-transformed. Note that when LMMs are run on ln-transformed data, post hoc test results are reported here on the ln scale, while they are given on the response scale in the main text to ease communication.

Residuals normality and homoscedasticity are checked graphically with the quantile-quantile plot and the latter by plotting the absolute residuals versus the model's predictions.

In some analyses, LMM assumptions are not complied with even after ln-transforming the response variable. However, it should be stressed that in these cases, the ln-transformation did improve the normality and the homoscedasticity of the residuals. In addition, LMMs are robust to some amount of departure from normality and some amount of heteroscedasticity.

**List of abbreviations**

- contrast: post hoc test paired comparison;
- DenDF: denominator degrees of freedom;
- df: degrees of freedom;
- emmean: estimated marginal mean;
- estimate: estimated groups difference for the contrast;
- lower.CL: lower confidence limit;
- Mean Sq: mean square;
- NumDF: numerator degrees of freedom;
- p.value: type 1 error probability;
- Pr(>F): type 1 error probability;
- SE: standard error;
- session: assessment session before (T0) vs after (T1) rehabilitation;
- STW: sit to walk phase of the TUG test;
- Sum Sq: sum of square;
- t.ratio: t-statistics;
- trial: for each variable, two measures have been collected in each session (Trial_2-3_ vs Trial_3-4_); data from the first habituation repetition are also included as a third level of this factor;
- TUG test: Timed Up and Go test;
- upper.CL: upper confidence limit;
- ω: peak angular velocity along the vertical axis during turning.

***Within- and between-sessions time course of the mobility measurements.***

**Gait speed**

Type III Analysis of Variance Table with Satterthwaite's method

**Sum Sq Mean Sq NumDF DenDF F value Pr(>F)**

session 6.4342 6.4342 1 979.01 482.4839 <2e-16 ***

trial 1.4080 0.7040 2 979.01 52.7926 <2e-16 ***

session:trial 0.0088 0.0044 2 979.01 0.3316 0.7178


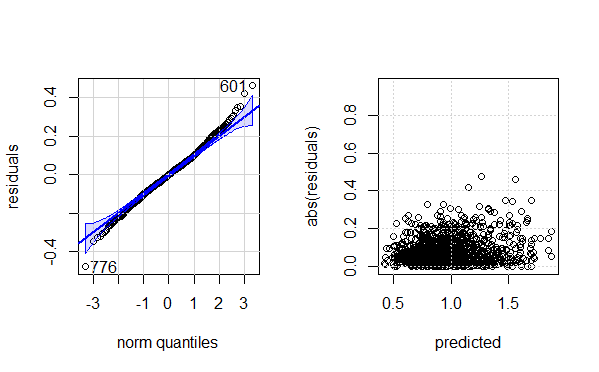


Left: quantile-quantile plot; right: absolute residuals vs model's predicted values. The two most extreme observations are labelled in the quantile-quantile plot.

**session emmean SE df lower.CL upper.CL**

T0 0.898 0.0261 114 0.846 0.949

T1 1.060 0.0261 114 1.008 1.111

Results are averaged over the levels of: trial

Degrees-of-freedom method: satterthwaite

Confidence level used: 0.95

**trial emmean SE df lower.CL upper.CL**

0 0.923 0.0267 126 0.870 0.975

1 0.992 0.0261 115 0.941 1.044

2 1.021 0.0261 115 0.969 1.073

Results are averaged over the levels of: session

Degrees-of-freedom method: satterthwaite

Confidence level used: 0.95

**contrast estimate SE df t.ratio p.value**

1 - 2 0.0696 0.00958 979 7.266 <.0001

1 - 3 0.0984 0.00959 979 10.259 <.0001

2 - 3 0.0287 0.00783 979 3.671 0.0003

Results are averaged over the levels of: session

Degrees-of-freedom method: satterthwaite

P value adjustment: holm method for 3 tests

**TUG test duration**

Type III Analysis of Variance Table with Satterthwaite's method

**Sum Sq Mean Sq NumDF DenDF F value Pr(>F)**

session 1372.61 1372.61 1 981 351.1146 <2e-16 ***

trial 315.28 157.64 2 981 40.3245 <2e-16 ***

session:trial 15.18 7.59 2 981 1.9411 0.1441


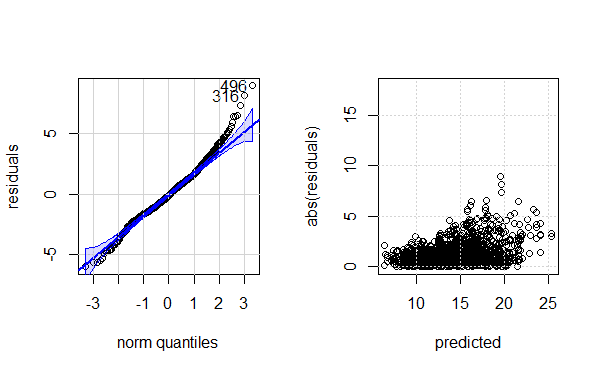


**TUG test duration, ln-transformed**

Type III Analysis of Variance Table with Satterthwaite's method

**Sum Sq Mean Sq NumDF DenDF F value Pr(>F)**

session 5.8449 5.8449 1 981 382.877 <2e-16 ***

trial 1.3400 0.6700 2 981 43.890 <2e-16 ***

session:trial 0.0201 0.0100 2 981 0.658 0.5181


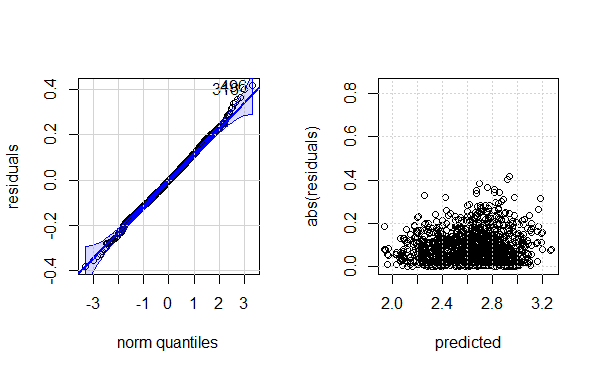


**session emmean SE df lower.CL upper.CL**

T0 2.71 0.0249 115 2.66 2.76

T1 2.55 0.0249 115 2.51 2.60

Results are averaged over the levels of: trial

Degrees-of-freedom method: satterthwaite

Results are given on the log (not the response) scale.

Confidence level used: 0.95

**trial emmean SE df lower.CL upper.CL**

0 2.69 0.0256 130 2.64 2.74

1 2.62 0.0249 117 2.57 2.67

2 2.59 0.0249 117 2.54 2.64

Results are averaged over the levels of: session

Degrees-of-freedom method: satterthwaite

Results are given on the log (not the response) scale.

Confidence level used: 0.95

**contrast estimate SE df t.ratio p.value**

1 - 2 -0.0680 0.01025 981 -6.633 <.0001

1 - 3 -0.0959 0.01025 981 -9.354 <.0001

2 - 3 -0.0279 0.00837 981 -3.333 0.0009

Results are averaged over the levels of: session

Degrees-of-freedom method: satterthwaite

Results are given on the log (not the response) scale.

P value adjustment: holm method for 3 tests

**STW duration**

Type III Analysis of Variance Table with Satterthwaite's method

**Sum Sq Mean Sq NumDF DenDF F value Pr(>F)**

session 0.57899 0.57899 1 957.17 5.9694 0.01474 *

trial 0.04185 0.02093 2 949.82 0.2158 0.80597

session:trial 0.15886 0.07943 2 949.52 0.8189 0.44123
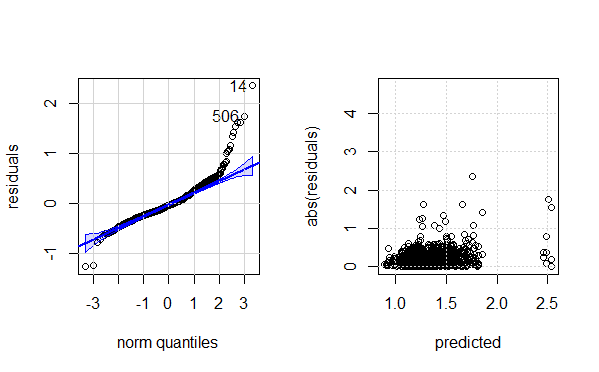


**STW duration, ln-transformed**

Type III Analysis of Variance Table with Satterthwaite's method

**Sum Sq Mean Sq NumDF DenDF F value Pr(>F)**

session 0.202150 0.202150 1 955.49 4.7878 0.0289 *

trial 0.021518 0.010759 2 948.36 0.2548 0.7751

session:trial 0.065470 0.032735 2 948.07 0.7753 0.4609
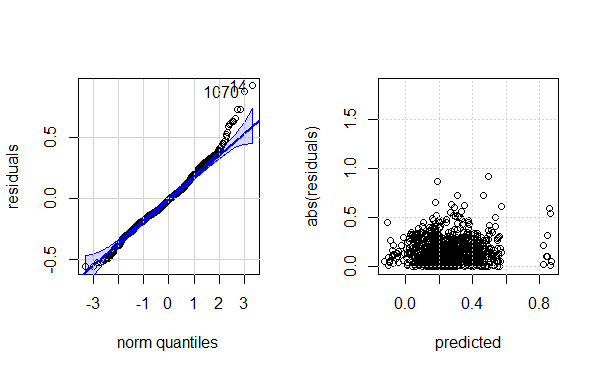


**session emmean SE df lower.CL upper.CL**

T0 0.266 0.0178 156 0.231 0.302

T1 0.237 0.0176 149 0.202 0.272

Results are averaged over the levels of: trial

Degrees-of-freedom method: satterthwaite

Results are given on the log (not the response) scale.

Confidence level used: 0.95

**Turning duration**

Type III Analysis of Variance Table with Satterthwaite's method

**Sum Sq Mean Sq NumDF DenDF F value Pr(>F)**

session 9.4241 9.4241 1 954.46 32.5289 1.566e-08 ***

trial 1.2955 0.6477 2 950.56 2.2358 0.1075

session:trial 0.8163 0.4082 2 950.41 1.4088 0.2449
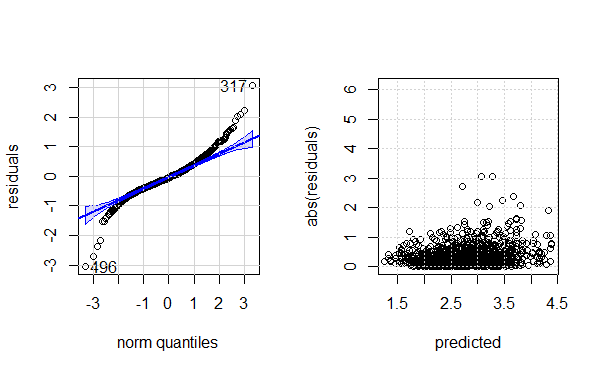


**Turning duration, ln-transformed**

Type III Analysis of Variance Table with Satterthwaite's method

**Sum Sq Mean Sq NumDF DenDF F value Pr(>F)**

session 1.00676 1.00676 1 952.09 27.0321 2.449e-07 ***

trial 0.29185 0.14592 2 948.56 3.9182 0.0202 *

session:trial 0.18624 0.09312 2 948.43 2.5003 0.0826
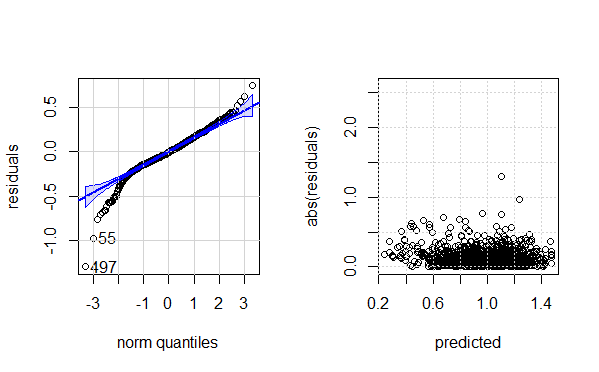


**session emmean SE df lower.CL upper.CL**

T0 0.999 0.0243 129 0.951 1.047

T1 0.933 0.0242 126 0.886 0.981

Results are averaged over the levels of: trial

Degrees-of-freedom method: satterthwaite

Results are given on the log (not the response) scale.

Confidence level used: 0.95

**trial emmean SE df lower.CL upper.CL**

0 0.958 0.0262 172 0.907 1.01

1 0.952 0.0244 132 0.904 1.00

2 0.988 0.0244 131 0.940 1.04

Results are averaged over the levels of: session

Degrees-of-freedom method: satterthwaite

Results are given on the log (not the response) scale.

Confidence level used: 0.95

**contrast estimate SE df t.ratio p.value**

1 - 2 -0.00605 0.0163 949 -0.372 0.7103

1 - 3 0.02962 0.0163 948 1.819 0.1384

2 - 3 0.03567 0.0133 949 2.684 0.0222

Results are averaged over the levels of: session

Degrees-of-freedom method: satterthwaite

Results are given on the log (not the response) scale.

P value adjustment: holm method for 3 tests

**Peak angular velocity along the vertical axis during turning (ω)**

Type III Analysis of Variance Table with Satterthwaite's method

**Sum Sq Mean Sq NumDF DenDF F value Pr(>F)**

session 25910.5 25910.5 1 952.32 91.6811 <2e-16 ***

trial 1105.3 552.6 2 950.28 1.9555 0.1421

session:trial 58.5 29.2 2 950.20 0.1034 0.9017
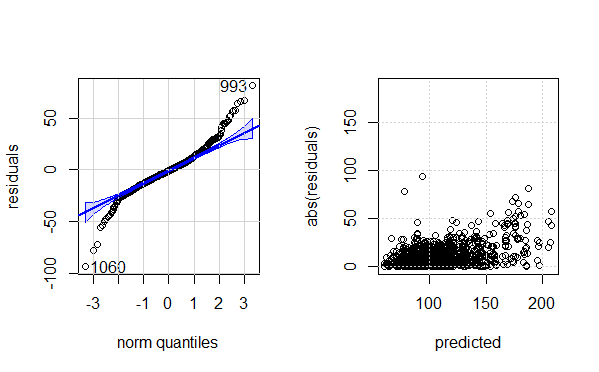


**ω, ln-transformed**

Type III Analysis of Variance Table with Satterthwaite's method

**Sum Sq Mean Sq NumDF DenDF F value Pr(>F)**

session 1.94634 1.94634 1 950.01 111.5208 <2e-16 ***

trial 0.04331 0.02165 2 948.25 1.2408 0.2896

session:trial 0.00500 0.00250 2 948.18 0.1434 0.8665


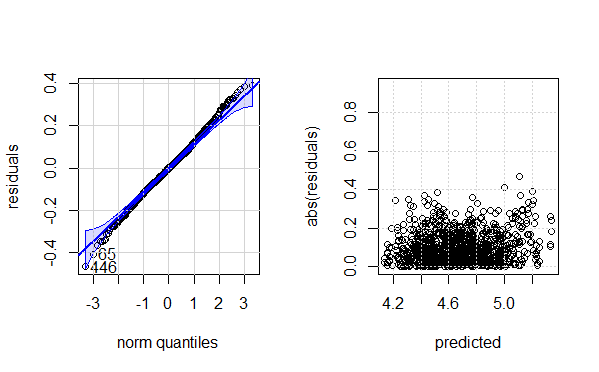


**session emmean SE df lower.CL upper.CL**

T0 4.61 0.0238 118 4.57 4.66

T1 4.71 0.0237 117 4.66 4.75

Results are averaged over the levels of: trial

Degrees-of-freedom method: satterthwaite

Results are given on the log (not the response) scale.

Confidence level used: 0.95

***Estimating SEM: compliance with the LMMs assumptions.***

| **Gait speed** | **Gait speed, ln-transformed** |
| --- | --- |
| 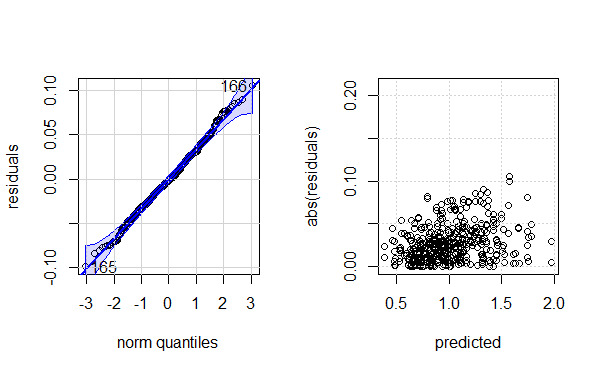 | 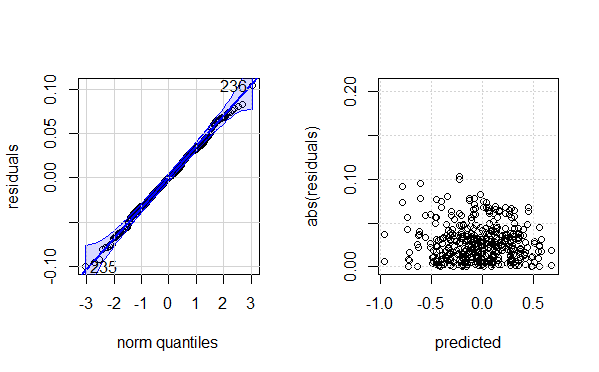 |
| **TUG test duration** | **TUG test duration, ln-transformed** |
| 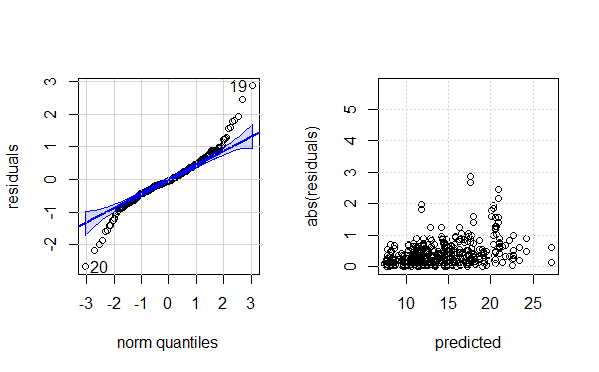 | 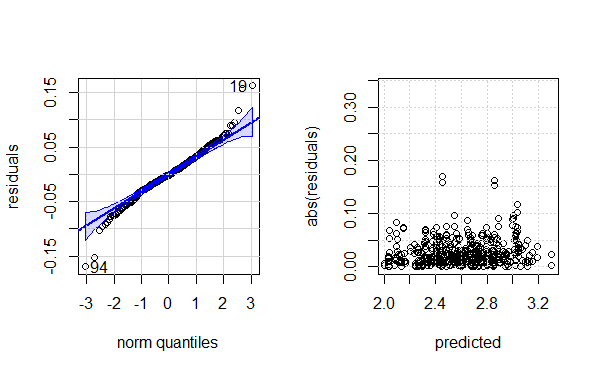 |
| **STW duration** | **STW duration, ln-transformed** |
| 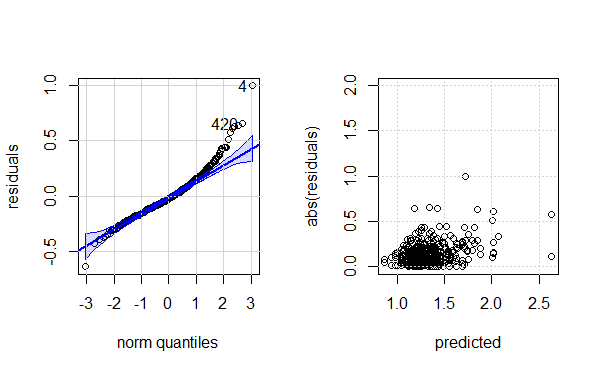 | 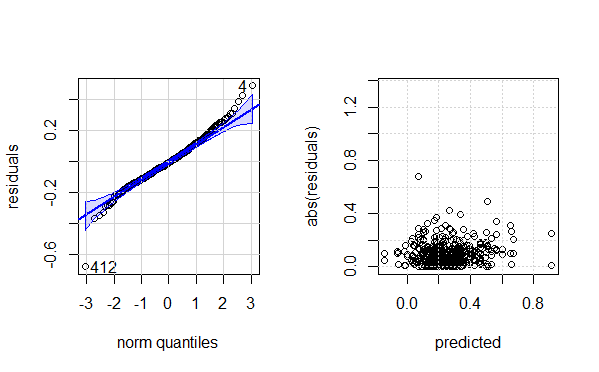 |
|  |  |
| **Turning duration** | **Turning duration, ln-transformed** |
| 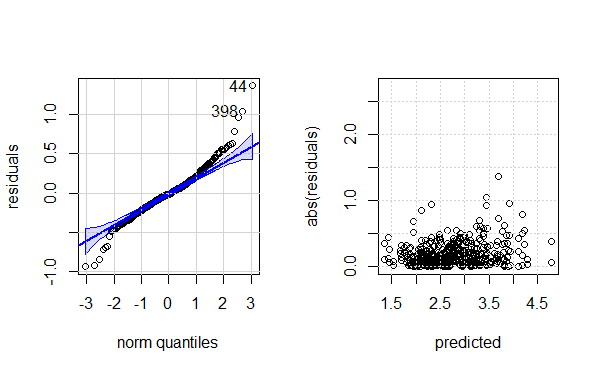 | 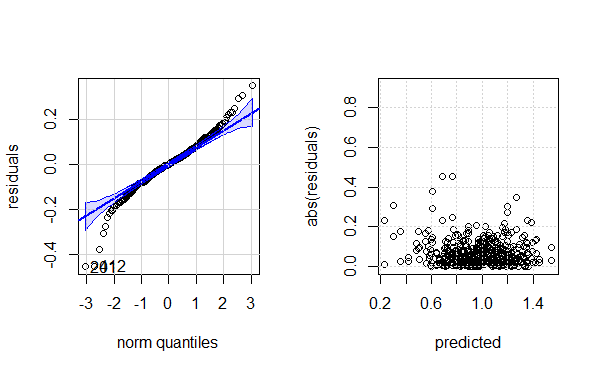 |
| **ω** | **ω, ln-transformed** |
| 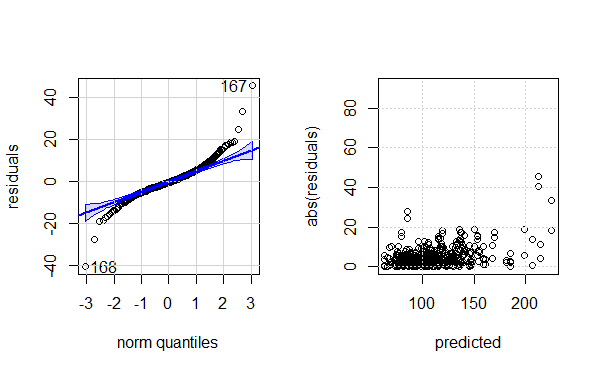 | 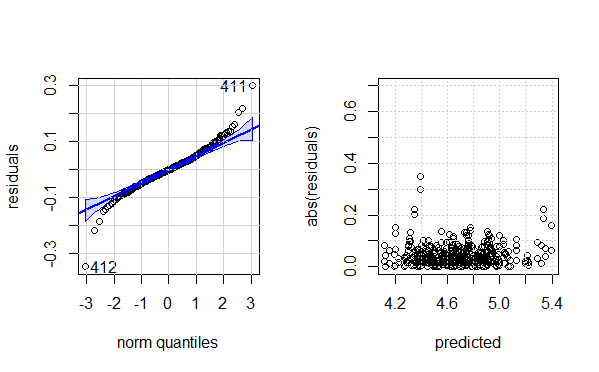 |
